# Anti-CD20 Immunoglobulin G Radiolabeling with a ^99m^Tc-Tricarbonyl Core: *In Vitro* and *In Vivo* Evaluations

**DOI:** 10.1371/journal.pone.0139835

**Published:** 2015-10-06

**Authors:** Hélène Carpenet, Armelle Cuvillier, Jacques Monteil, Isabelle Quelven

**Affiliations:** 1 Nuclear Medicine Department, Dupuytren University Hospital, Limoges, France; 2 EA 3842, Faculty of Medicine and Pharmacy, Limoges, France; 3 B Cell Design Society, Limoges, France; Vrije Universiteit Brussel, BELGIUM

## Abstract

In recent years, the diagnostic and therapeutic uses of radioisotopes have shown significant progress. Immunoglobulin (Ig) appears to be a promising tracer, particularly due to its ability to target selected antigens. The main objective of this study is to optimize and assess an Ig radiolabeling method with Technetium 99m (^99m^Tc), an attractive radioelement used widely for diagnostic imaging. Monoclonal anti-CD20 IgG was retained to study *in vitro* and *in vivo* radiolabeling impact. After IgG derivatization with 2-iminothiolane, IgG-SH was radiolabeled by an indirect method, using a ^99m^Tc-tricarbonyl core. Radiolabeling stability was evaluated over 24h by thin-layer chromatography. IgG integrity was checked by sodium dodecyl sulfate—polyacrylamide gel electrophoresis coupled with Western blot and autoradiography. The radiolabeled Ig’s immunoaffinity was assessed *in vitro* by a radioimmunoassay method and binding experiments with cells (EL4-hCD20 and EL4-WT). Biodistribution studies were performed in normal BALB/c mice. Tumor uptake was assessed in mice bearing EL4-hCD20 and EL4-WT subcutaneous xenografts. With optimized method, high radiolabeling yields were obtained (95.9 ± 3.5%). ^99m^Tc-IgG-SH was stable in phosphate-buffered saline (4°C and 25°C) and in serum (37°C), even if important sensitivity to transchelation was observed. IgG was not degraded by derivatization and radiolabeling, as shown by Western blot and autoradiography results. ^99m^Tc-anti-CD20 IgG-SH immunoaffinity was estimated with Kd = 35 nM by both methods. *In vivo* biodistribution studies for 48h showed significant accumulation of radioactivity in plasma, liver, spleen, lungs and kidneys. Planar scintigraphy of mice bearing tumors showed a significant uptake of ^99m^Tc-anti-CD20 IgG-SH in CD20^+^ tumor versus CD20^-^ tumor. Radiolabeling of derivatized IgG with ^99m^Tc-tricarbonyl was effective, stable and required few antibody amounts. This attractive radiolabeling method is “antibody safe” and preserves Ig affinity for antigen, as shown by both *in vitro* and *in vivo* experiments. This method could easily be used with noncommercial IgG or other antibody isotypes.

## Introduction

Due to their highly specific targeting ability, monoclonal antibodies and their fragments are considered attractive candidates to deliver radioelements to an interest target (particularly in oncology). The aim of this work was to optimize, a convenient and non-damaging Ig radiolabeling method that can be applied to Ig fragments and different isotypes. The radiolabeling method should provide efficient yields while maintaining antibody structure and functionality. To assess the radiolabeling process in the study, the IgG isotype and technetium 99m (^99m^Tc) were selected, due to their widespread use and ready accessibility. Among the radionuclides used in diagnosis, ^99m^Tc remains the most widely used isotope. This radionuclide has favorable decay properties (energy of 140 keV of pure γ-radiation, 6h half-life) for molecular imaging and radiation protection. Moreover, its daily availability from a generator system is also a great asset. However, the short decay half-life of ^99m^Tc is not ideal in following the entire process of IgG biokinetics, although this theoretical disadvantage has not prevented its human use. Today, only intact or fragments of monoclonal antibodies approved by the FDA (Food and Drug Administration) are radiolabeled with ^99m^Tc. Besilesomab, a chimeric monoclonal anti-NCA 95 antibody, and sulesomab, an anti-NCA–90 Fab’ fragment, are indicated for infection imaging [1,2]. Finally, another attractive property of ^99m^Tc lies in its polyvalent radiochemistry. Technetium chemistry is ruled by the formation of metal-ligand complexes. To produce these complexes, the technetium eluted from the generator (^99m^TcO_4_^-^) must be reduced to lower oxidation states. For radiolabeling antibodies with ^99m^Tc, several techniques have been proposed using direct or indirect synthesis pathways. *Direct antibody radiolabeling* can be performed after reduction of disulfide bridges [3]. This method’s drawbacks lie in the lower stability of the complex obtained and the high probability of structure alterations. *Indirect radiolabeling*, *via a bifunctional chelating agent* (BFCA), has been promoted by development of new synthons such as MAG3 [4] and HYNIC [5]. Among the BFCAs, “tricarbonyl core” [Tc(CO)_3_(H_2_0)_3_]^+^ has many advantages, e.g., permitting ^99m^Tc to link with proteins under mild physiological conditions [6,7]. Moreover, tricarbonyl core is in a low oxidation state (+ I), with a high kinetic stability. Biechlin *et al*. showed that this complex was much more stable than the HYNIC method in radiolabeling proteins [8]. Alberto *et al*. first developed the ^99m^Tc-tricarbonyl core [6]. ^99m^Tc pertechnetate must be reduced to the +I state by direct reduction using a strong and soluble reducing agent under carbon monoxide gas conditions. This first limited method of core production has been replaced by an easier and reproducible method using a commercial one-step kit formulation [9]. In this kit (IsoLink^®^, Mallinckrodt), boranocarbonate sodium allows both the production of carbon monoxide and the reduction of technetium. Different antibody radiolabeling strategies using ^99m^Tc-tricarbonyl have been assessed, with direct antibody labeling [7,10] or using a spacer [11]. Investigations by Egli *et al*. showed that histidine was the most potent ligand among other amino acids and led to direct radiolabeling of peptides or proteins [12]. Radiolabeling yields with direct approaches are often insufficient and supplementary steps such as purification are then required. A spacer may facilitate the addition of the metal ion to the protein. Among the spacers used to bind the tricarbonyl core, 2-iminothiolane (2-IT, Traut's reagent) has been studied [13]. 2-IT has the advantage of allowing the reaction under non-denaturing conditions. It reacts with free amino groups of Ig, to produce the mercaptobutyrimidyl group (MBG) and generates free thiol residues on Ig (Ig-SH). This reaction can increase the Ig reactivity against ^99m^Tc complexes and improve labeling yields [14]. Biechlin *et al*. demonstrated that the combination of ^99m^Tc-tricarbonyl with 2-IT derivatization could be applied to various proteins such as IgG with high yields [15], showing with polyvalent IgG that this radiolabeling was efficient and highly stable. However, the preservation of antibodies structure and affinity, after this two-step radiolabeling method has not been evaluated. Considering the various advantages of using a tricarbonyl core after Ig functionalization with 2-IT, we chose to apply and optimize this promising method. Radiolabeled antibody integrity and affinity were evaluated by *in vitro* and *in vivo* studies. To this aim, we selected a widely used monoclonal antibody, rituximab (commercialized as Rituxan^®^ in the United States and as Mabthera^®^ in Europe). Rituximab is a chimeric mouse/human IgG1 monoclonal antibody directed against the transmembrane antigen CD20. Rituximab binds the CD20 antigen with a high affinity (Kd = 5.2–11.0 nM) [16].

Thus, the purpose of the present study was to optimize an IgG radiolabeling method with ^99m^Tc. Functionalization of the antibody with 2-IT and complexation with ^99m^Tc-tricarbonyl core were applied to radiolabel anti-CD20 IgG. The radiolabeled IgG was then characterized by both *in vitro* and *in vivo* approaches to verify Ig function preservation.

## Materials and Methods

### Materials

All chemicals and reagents were obtained from Sigma-Aldrich (Saint-Quentin Fallavier, France), unless otherwise specified.

Anti-CD20 IgG (rituximab) and irrelevant IgG (anti-EGFR: panitumumab) were purified from Mabthera^®^ (Hoffmann-La Roche, Basel, Switzerland) and from Vectibix^®^ (Amgen, Breda, The Netherlands) respectively, by affinity chromatography. Subsequently, IgG was dialyzed against phosphate-buffered saline (PBS) by centrifugation (1000 x g, 15 min) using Amicon 30 kDa (Millipore, Molsheim, France).

Pertechnetate [Na^99m^TcO_4_] was eluted freshly from a ^99^Mo/^99m^Tc generator (CisBio, Saclay, France). IsoLink^®^ kit was obtained from Mallinckrodt Medical (Petten, The Netherlands).

ITLC-SG strips were purchased from Varian (Les Ulis, France) and Baker-Flex aluminium oxide strips were obtained from J.T.Baker Inc (VWR, Pessac, France). Protein concentrations and antibody quantity injected to mice were determined using bicinchoninic assay kits (Micro BC Assay^®^, Fisher Scientific, Illkirch, France). The CD20 antigen used for the radio-immuno-assay was synthesized and purified by Millegen (Toulouse, France). This antigen corresponds to CD20 extracellular region and disulfide bond has been conserved to form the most immunogenic loop according to Ernst's [17]. Electrophoresis reagents were from Bio-Rad (Marnes-La-Coquette, France) and cell culture media and supplements were from Invitrogen (Carlsbad, USA). Activity measurements were carried out using an activimeter Medi 405 (Medisystem, Guyancourt, France) and a gamma-counter (Cobra 5003; Packard Canberra Company, Frankfurt, Germany). Female BALB/c mice and female athymic immunodeficient nude mice were purchased from Charles River Laboratories (L’Arbresle, France).

### Thiol-derivatization of Ig with 2-IT

**Fig 1:**
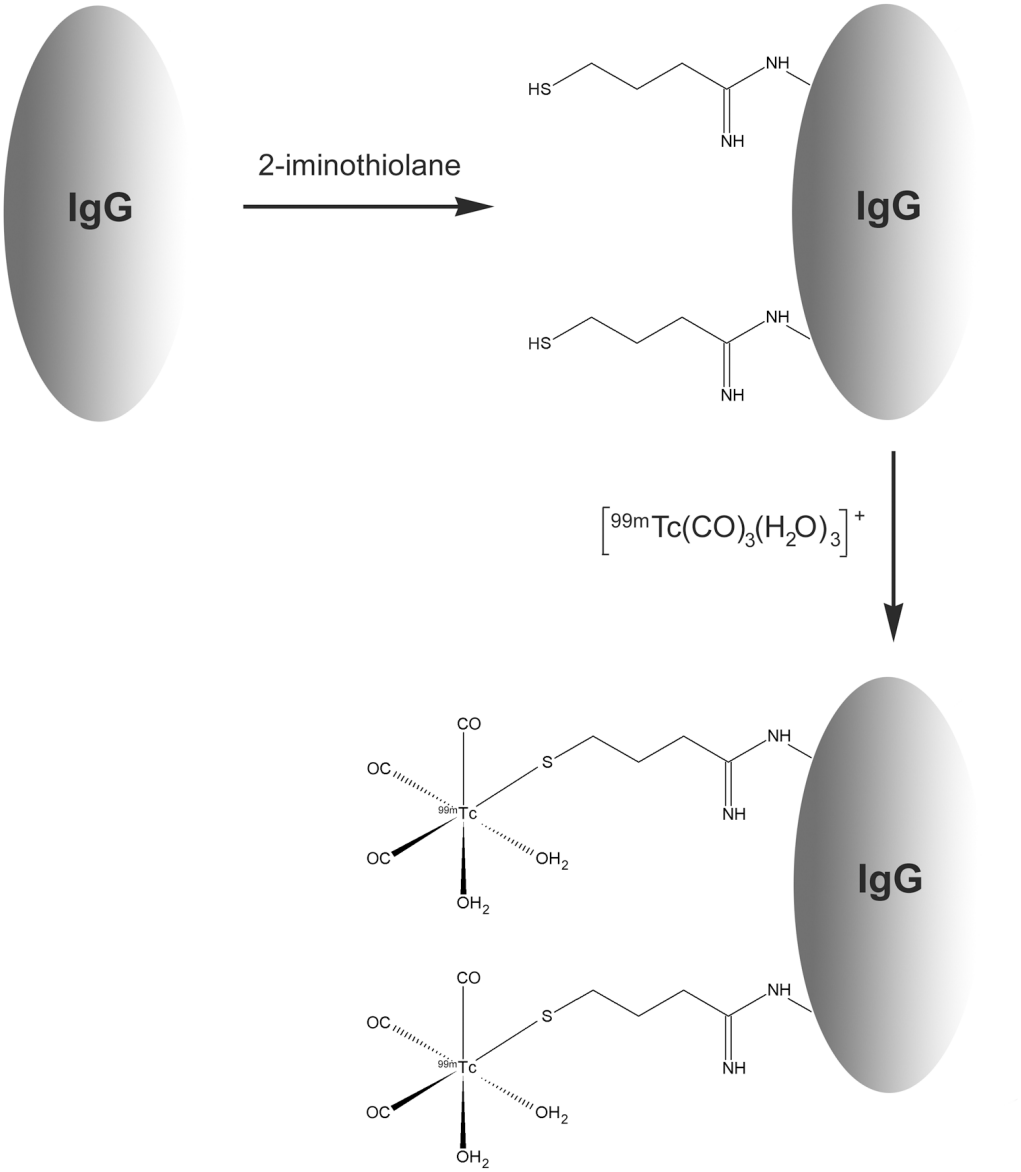
Two-step IgG radiolabeling. After IgG functionalization with 2-iminothiolane (2-IT), ^99m^Tc-tricarbonyl core [^99m^Tc(CO)_3_(H_2_0)_3_]^+^ preformed was complexed with IgG functionalized (IgG-SH).

First, 0.5–2 nmol IgG (200 μL in PBS) was incubated with 2-IT at a final concentration of 3.8 μM (Fig 1). The reaction proceeded at room temperature in a low-adsorption tube for 120 min, with stirring. After dilution with 100 μL of PBS, solutions were purified by size exclusion chromatography with Zeba Spin Desalting Columns (Fisher Scientific) after centrifugation (1000 × *g*, 2 min, 4°C).

The number of thiol groups was determined with a micromethod using Ellman’s reagent (5.5’-dithiobis-2-nitrobenzoic acid, DTNB) followed by a spectrophotometric assay at 405 nm. Cysteine was used as a standard (2.5–100 μM). Results are expressed as sulfhydryl groups generated per antibody molecule.

### Synthesis of the tricarbonyl precursor [^99m^Tc(CO)_3_(H_2_0)_3_]^+^

First, 0.8–1 mL of [Na^99m^TcO_4_] in fixed activities (2220–3700 MBq) was added to the IsoLink^®^ vial, incubated in a boiling water bath for 25 min and cooled in water for 10 min.

In each synthesis, a radiochemical purity (RCP) analysis was performed by thin-layer chromatography (TLC) with two systems and assessed with a radiochromatograph Miniscan^®^ from Bioscan:

1. System I (Baker Flex Aluminium oxide strips in methanol/hydrochloric acid (95/5 v/v)): Rf ([^99m^Tc(CO)_3_(H_2_0)_3_]^+^ and [^99m^Tc(OH)_n_(H_2_0) _y_]) = 0, Rf ([^99m^Tc]-pertechnetate) = 1
2. System II (ITLC-SG strips in methanol): Rf ([^99m^Tc(OH)_n_(H_2_0)_y_]) = 0, Rf ([^99m^Tc(CO)_3_(H_2_0)_3_]^+^ and [^99m^Tc]-pertechnetate) = 1

### Radiolabeling of native or derivatized immunoglobulins with [^99m^Tc(CO)_3_(H_2_0)_3_]^+^

^99m^Tc-tricarbonyl was neutralized to pH 7.0 using 0.5 M hydrochloric acid to perform Ig radiolabeling under non-denaturing conditions. In a low adsorption tube, 0.5–4 nmol of non-derivatized IgG or 0.5–2 nmol of derivatized IgG (IgG-SH) in 300 μL PBS was mixed with 150 μL (148–185 MBq) of a ^99m^Tc-tricarbonyl solution (Fig 1). Each sample was incubated at room temperature for 120 min with stirring.

The RCP was determined by ITLC-SG/NaCl 0.9%: Rf ([^99m^Tc]-Ig or ([^99m^Tc]-Ig-SH) = 0 and Rf ([^99m^Tc(CO)_3_(H_2_0)_3_]^+^) = 1.

### *In vitro* stability of radiolabeled immunoglobulins

Samples of radiolabeled antibody were incubated for 24 h in PBS at two different temperatures (4°C and 25°C) or in murine plasma (1/10) at 37°C. To determine their stability against transchelation and/or degradation, radiolabeled IgG was also tested at 37°C for 24 h in the presence of cysteine (7.5-, 15-, 150-, or 1500-fold excess over the number of thiol groups present in the radiolabeled IgG solution) or histidine (1500-fold excess). For each sample, aliquots were analyzed by ITLC at 1 h, 2 h, 4 h, 16 h, and 24 h.

### Sodium dodecyl sulfate polyacrylamide gel electrophoresis (SDS-PAGE) for Western blot or autoradiography

SDS-PAGE was performed under nonreducing conditions with the native or labeled Ig. Proteins (1.5–10 μg per lane) and molecular weight standards (Bio-Rad) were loaded and resolved with a Mini-Protean TGX Precast Gel using a Bio-Rad (Hercules, CA, USA) apparatus. One gel was used for radioactivity measurements using a phosphor screen (Multisensitive Medium MS; Perkin Elmer, Courtaboeuf, France) revealed by an autoradiochromatograph scanner (Cyclone Storage Phosphor System; Perkin Elmer). A duplicate gel was transferred on a PVDF membrane. Membrane was incubated (90 minutes) in diluted primary antibody solution (1/1000, goat antihuman IgG-horse radish peroxidase; Southern Biotech, Birmingham, AL, USA) and revealed with a diaminobenzidine (DAB) substrate.

### Binding affinity of radiolabeled Ig by radioimmunoassay (RIA)

To evaluate anti-CD20 IgG binding to human CD20, removable well plates were coated with CD20 at 1 μg/well and incubated at 4°C overnight in PBS (pH 7.4), then blocked with PBS-gelatin 2% for 1 h at 37°C. Radiolabeled Ig-relevant (anti-CD20 IgG) or irrelevant (anti-EGFR IgG) (100 μl, 0,5–32 μg/mL) was added in two series to antigen-coated wells. The first series was diluted in PBS-gelatin 0.2% (total binding) and the second in PBS-gelatin 0.2% containing unlabeled antibody at 500 μg/mL (non-specific binding). Plates were incubated for 2 h at 37°C and were washed three times with PBS. Radioactivity contained in the wells was determined by gamma counting.

### Cell culture

Murine lymphoma T-cells line not expressing human CD20 (EL4-WT) and murine lymphoma T-cells expressing human CD20 (EL4-hCD20) were gifts from UMR-CNRS 7276 (Prof. Michel Cogné, Limoges University, France) [18]. They were cultured in Dubelcco’s Modified Eagle Medium (DMEM) high glucose, buffered with HEPES, supplemented with 10% fetal calf serum, 1% nonessential amino acids, 1% sodium pyruvate, 1% penicillin (100 U/mL)—streptomycin (100 μg/mL), *β*-mercaptoethanol (9 × 10^−4^%) and neomycin (800 μg/mL, 0.1%) for EL4-hCD20 cells only. The level of CD20 expression was checked regularly by flow cytometric analysis, using a murine FITC-labeled anti-human CD20 IgG (Dako, Glostrup, Denmark).

### Binding affinity of radiolabeled Ig on cells

Saturation binding experiments with ^99m^Tc-Ig-SH were performed with EL4-hCD20 and EL4-WT cells for anti-CD20 IgG. With EL4-hCD20 cells, anti-EGFR IgG was used as an irrelevant antibody control. Samples of cells (1 millions cells/ 250 μl), pre-incubated (90 min, 25°C) with unlabeled antibody (final concentration of 100 μg/mL) or with PBS-BSA 3%, were incubated with increasing concentrations of ^99m^Tc-IgG-SH (125 μL, 0,23–15 μg/mL). After incubation (2 h, 25°C), the cells were filtered using Manifold^®^ (Millipore, Molsheim, France) and washed with PBS. Filter radioactivity was evaluated with a gamma-counter.

### Affinity data analysis

For wells and cell-binding affinity studies, specific binding was evaluated by subtracting, nonspecific binding (determined after incubation with unlabeled antibody) from total binding. Data analysis was performed using a Scatchard plot of binding of ^99m^Tc-anti-CD20-IgG-SH and the dissociation constant (Kd), the number of antibody binding sites/wells or cells, and the Bmax of ^99m^Tc-anti-CD20 IgG-SH were determined.

### Antibody immunoreactivity

The immunoreactivity fraction (IRF) was assessed according to the method of Lindmo *et al*. [19,20]. An immunoreactivity study was performed in microtubes using increasing concentrations of EL4-hCD20 cells (0,16 x 10^6^–10 x 10^6^ cells in 250 μL of 3% PBS-BSA). Nonspecific binding was determined by 1 h previous incubation of one series of cell dilutions with unlabeled antibody (100 μg/mL). ^99m^Tc-anti-CD20 IgG-SH (125 μL, 800 ng/mL) was added to each tube containing the cell dilutions. After incubation (2 h, 25°C), the cells were filtered using Manifold^®^ and washed with PBS. Cell-bound radioactivity was determined by measuring filters with a gamma-counter. The data were plotted as a double inverse plot of specific binding over total applied radioactivity as a function of increasing cell concentration.

### Biodistribution of ^99m^Tc-anti-CD20 IgG-SH in normal mice

All *in vivo* experiments were performed in accordance with animal ethical regulations and all efforts were made to minimize suffering. The protocol was approved by Comité Régional d'Ethique sur l'Expérimentation Animale du Limousin (CREEAL). Biodistribution experiments were carried out in 7-week-old male BALB/c mice (Charles River Laboratories). ^99m^Tc-anti-CD20 IgG-SH (40 MBq, 170 μg of antibody) was injected intravenously (tail vein). Animals were euthanized, by anesthesia and cervical dislocation, at 4 h (n = 3), 8 h (n = 4), 18 h (n = 4), 24 h (n = 7) or 48 h (n = 4) after injection. Selected tissues were excised, rinsed and weighed and their radioactivity levels were measured with a gamma-counter. Uptake of radioactivity in these organs was expressed as a percentage of the injected dose per gram of tissue (% ID/g) after correcting for radioactive decay for each time point.

### Tumor uptake of ^99m^Tc-anti-CD20 IgG-SH

Seven-week-old male nude mice were injected subcutaneously with 5 x 10^6^ EL4-hCD20 cells in one flank and with 2 x 10^7^ EL4-WT cells in the other, to obtain comparable tumors size. Animals were used for experiments when tumors size reached 0.9 to 1.1 cm at the largest diameter (4–6 weeks post cells injection). If animals demonstrated clinical alteration or tumor size exceeded 1.2 cm at the largest diameter, animals were euthanized, by anesthesia and cervical dislocation. Animals were divided into three experimental groups. The first group (n = 6) received 170 μg ^99m^Tc-anti-CD20 IgG-SH intravenously, the second (n = 3) received 170 μg ^99m^Tc-anti-EGFR IgG-SH and the third, the control group (n = 3) received only ^99m^Tc-IsoLink^®^. All the mice were subjected to an activity of 35–37 MBq. Scintigraphy imaging studies were performed 2 h 30, 12 h and 24 h after injection for ^99m^Tc-anti-CD20 IgG-SH animals groups and 24 h post-injection for ^99m^Tc-anti-EGFR IgG-SH and ^99m^Tc-IsoLink^®^ group. Mice were anesthetized using isoflurane (2%) under O_2_ conditions (1 L/min) and acquisitions were performed with a gamma camera (Axis; Philips Medical Systems, Cleveland, OH, USA). The planar images (256 x 256 pixels, zoom 2) were acquired during 10 minutes, using a dual-head system fitted with low-energy collimators. Energy windows were set over the 140 keV peaks (±20%). A region of interest (ROI) analysis was carried out using the Odyssey FX 820 software to determine tumor uptake. The semiquantitative uptake was calculated using the tumor to normal tissue background ratio. Muscle (upper limb) was used as normal tissue reference. Statistical analyses were made by applying the nonparametric Wilcoxon paired for kinetic uptake analysis and Mann-Whitney *U*-test for treatment comparison. Tests were made using the SAS software (ver. 9.1.3; SAS Institute, Cary, NC, USA). A *p* value < 0.05 was considered to indicate statistical significance.

## Results

### Radiolabeling optimization

**Fig 2:**
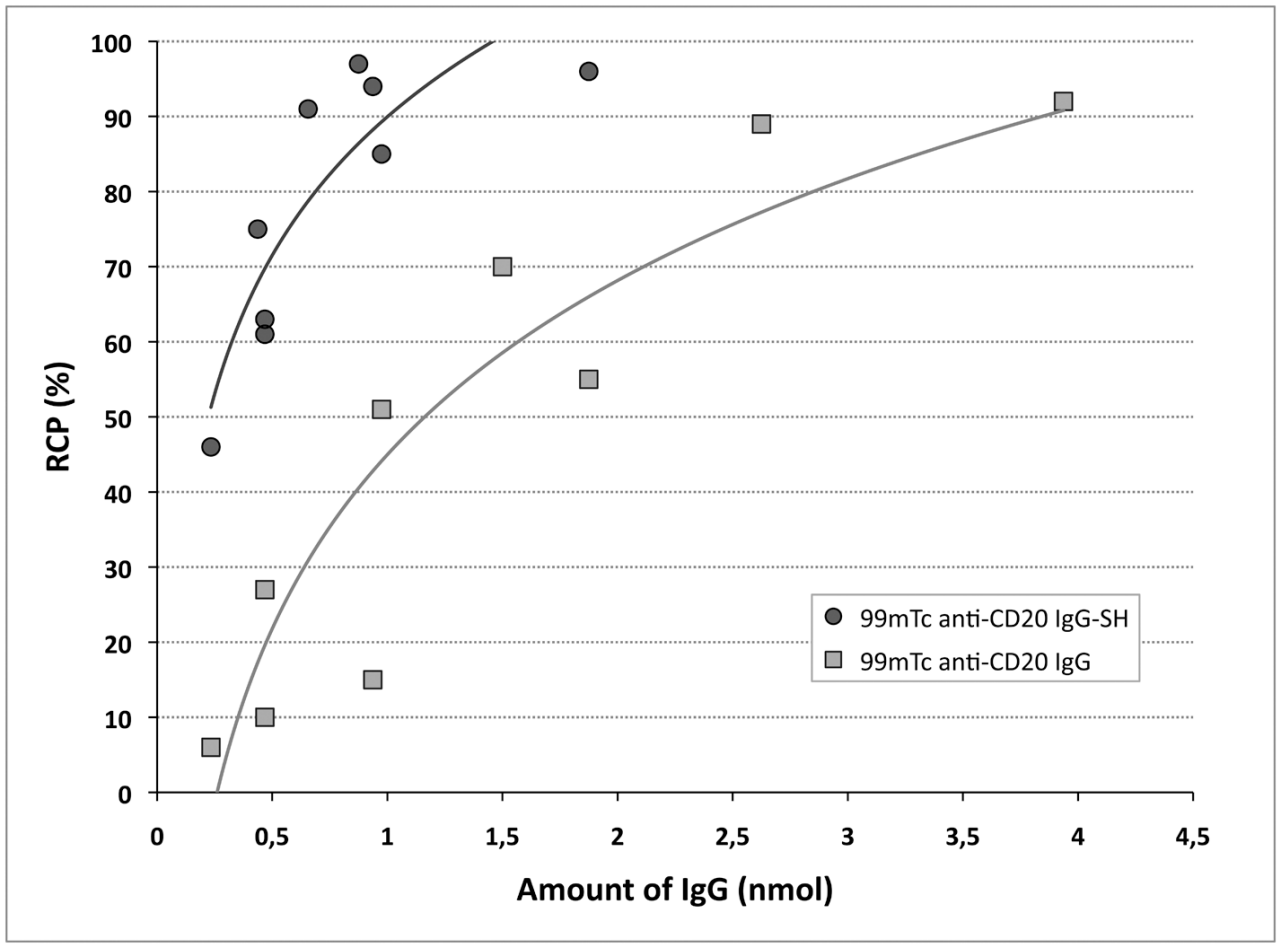
Comparison of radiochemical yields obtained with native or 2-IT derivatized IgG, depending on IgG amount. Radiochemical purity (RCP) were performed by ITLC-SG/NaCl 0,9%.

**Fig 3:**
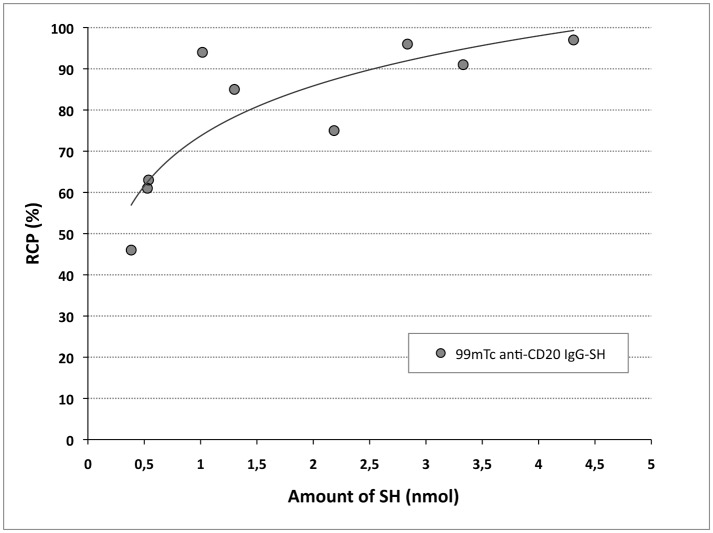
Radiolabeling yields of derivatized IgG as a function of SH group amount. Radiochemical purity (RCP) were performed by ITLC-SG/NaCl 0,9%.

Under the conditions described, the ^99m^Tc-IsoLink^®^ labeling yields were, on average, 98.8 ± 1.6% (n = 8). To optimize IgG radiolabeling with the tricarbonyl core, increasing antibody quantities and derivatization with 2-IT were studied. Native IgG radiolabeling yields obtained were correlated with IgG amounts and found to reach 90% with IgG amounts > 4 nmol (Fig 2). When IgG was functionalized by 2-IT, an RCP > 90% was obtained with only 1 nmol and a correlation between RCP and IgG amounts was observed. For the same IgG quantity, much higher yields were achieved if the Ig was derivatized: for 1 nmol IgG, the RCP was 86% versus 45% and for 0.5 nmol IgG, the RCP was 70% versus 20%, after derivatization and without respectively. Derivatization allowed reducing the IgG amount needed by a factor of 4 to obtain high radiolabeling yields. Determining sulfhydryl groups demonstrated that yields were clearly correlated with the number of thiol moieties on IgG (Fig 3). On average, 2.5 ± 1.4 (n = 8) sulfhydryl groups were grafted per molecule. In comparison with native IgG, sulfhydryl groups were below the limit of quantification. These results led us to use 1.5 nmol of derivatized IgG in the following experiments. Under these optimized conditions, radiolabeling yields were > 95.9 ± 3.5% (n = 8) and the specific activity corresponded to 123 MBq/nmol (822 MBq/mg).

### *In vitro* stability of radiolabeled IgG-SH

Stability of radiolabeled IgG studies were performed in different conditions during 24 h (Table 1). In PBS, with storage at +4°C or +25°C, no appreciable loss in RCP of ^99m^Tc-IgG-SH was observed (Δ = 2 and 3%, respectively). In murine serum at 37°C, the RCP decreased slightly to 82.3% at 24h, suggesting satisfactory stability *in vivo*.

**Table 1:** *In vitro* stability studies of derivatized radiolabeled IgG.

	T0	T+1h	T+2h	T+4h	T+16h	T+24h
**In PBS (+4°C)**	99	99	99	99	99	97
** (+25°C)**	99	99	99	99	96,7	96
**In murine serum (+37°C) (1:10)**	99	95,9	92	91,3	88,2	82,3
**+ Cysteine (+37°C) (7,5-fold)**	99	96	96	96	96	94
** (15-fold)**	99	95	89	83	76	74
** (150-fold)**	99	75	44	40	28	19
** (1500-fold)**	99	9	-	-	-	-
**+ Histidine (+37°C) (1500-fold)**	99	94	86	74	46	40

To assess the susceptibility of radiolabeled IgG to transchelation, in a physiological medium rich in plasma proteins with N-containing amino acids, studies in the presence of strong tridentate ligand systems (cysteine and histidine) were carried out. As expected, the RCP decreased depending on the nature and concentration of the competitor. Indeed, radiolabeled IgG degradation increased gradually as the cysteine excess increased: with 7.5-fold and 15-fold excesses, the RCP decreased by 5 and 25% respectively. With a 150-fold excess, the RCP dropped 80%, in 24 h. The 1500-fold excess of the number of thiol groups strongly challenged IgG in 1 h (Table 1). At same concentration (x 1500), radiolabeled IgG was only slightly degraded in the presence of histidine (RCP > 94% after 1 h).

### Integrity of radiolabeled antibodies

**Fig 4:**
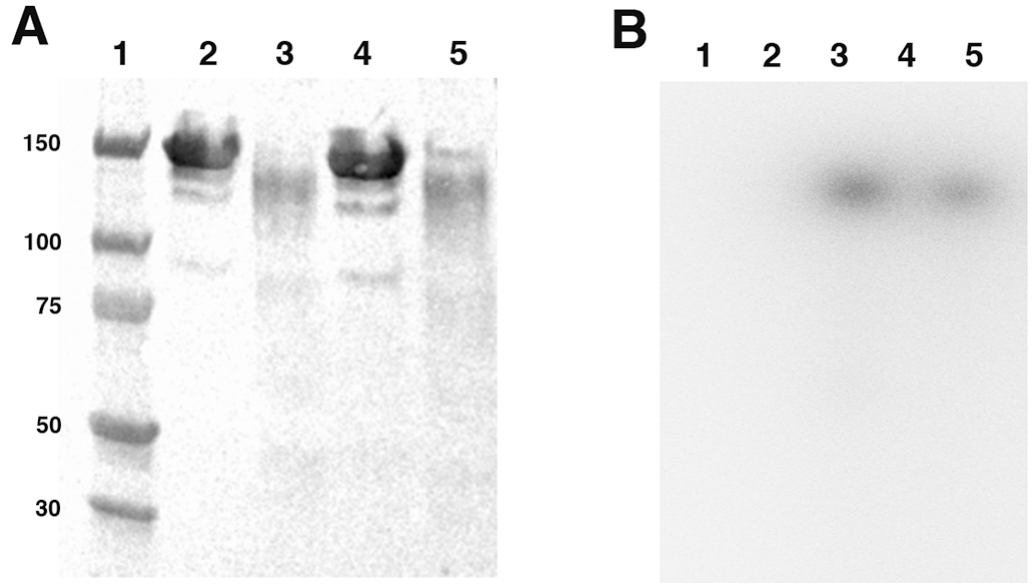
Western-blot (A) and autoradiography (B) of unlabeled and radiolabeled IgG. Molecular weights (kDa) are specified on the left. Well numbers (on the top) correspond to: (1) Marker, (2) Unlabeled anti-CD20 IgG (10μg), (3) Radiolabeled anti-CD20 IgG-SH (25μg), (4) Unlabeled irrelevant (anti-EGFR) IgG (10μg), (5) Radiolabeled irrelevant (anti-EGFR) IgG-SH (25μg).

Western blot profiles are presented in Fig 4A. Major bands were visualized at 150 kDa corresponding to the molecular weight of unlabeled IgG (lanes 2–4). It should be noted that weaker molecular weight bands, corresponding to degradation, were observed, but with very low intensity. Comparison between unlabeled and radiolabeled IgG (lines 3–5) showed that the homodimeric structure of IgG was preserved. However, a slight shift in migration and in intensity was observed between unlabeled and radiolabeled antibodies due to charges difference and to loss during purification after incubation with 2-IT, respectively.

The corresponding SDS-PAGE radioactivity profiles are presented in Fig 4B and show the same single band corresponding to 150 kDa for both radiolabeled IgGs. Comparison with the Western blot profiles of radiolabeled IgG (lanes 3–5) indicated that the radiolabeling occurred and remained linked to a fully folded IgG molecule.

### Immunoreactivity and binding affinity

**Fig 5:**
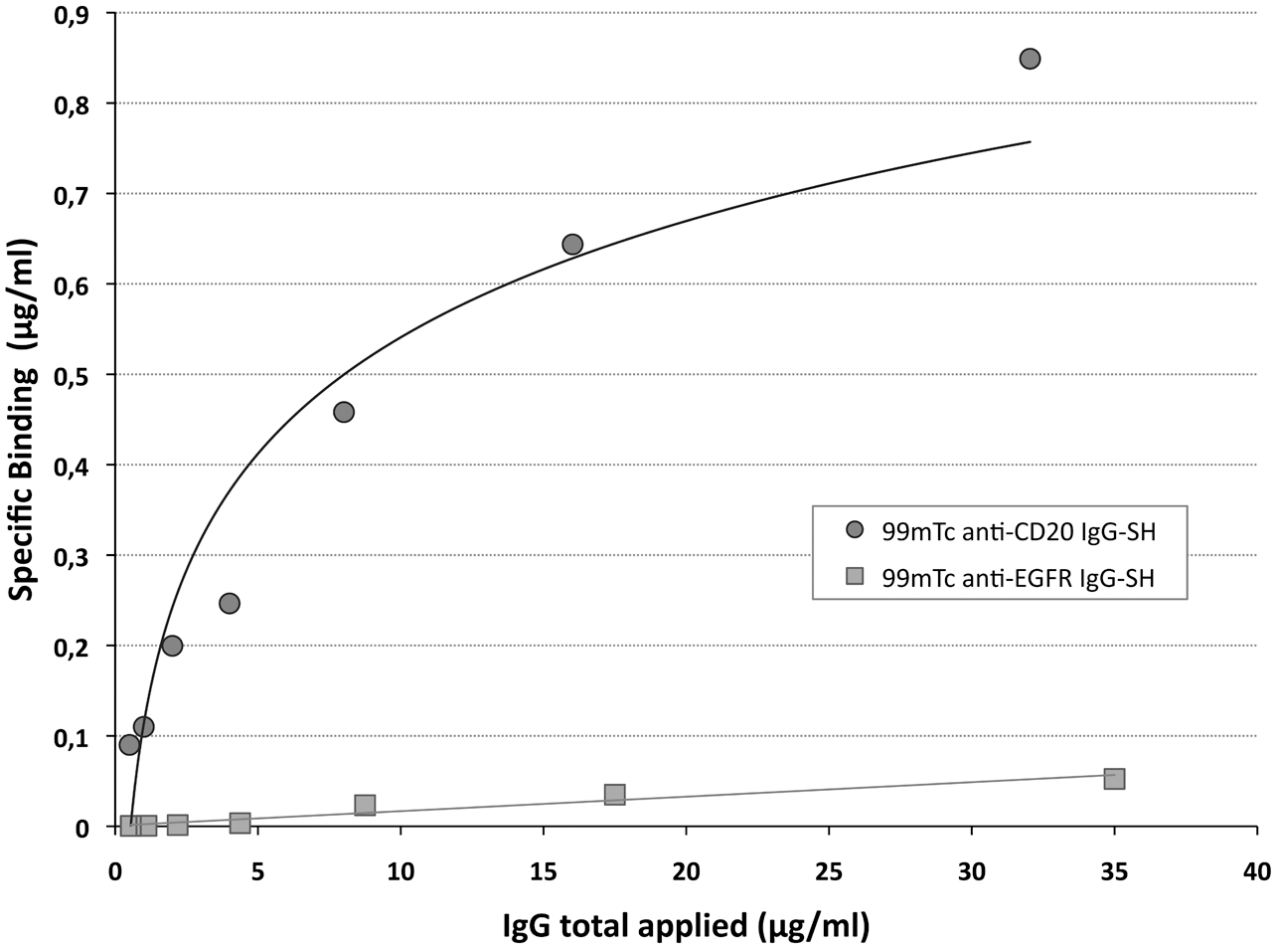
*In vitro* binding of ^99m^Tc anti-CD20 IgG-SH and ^99m^Tc anti-EGFR IgG-SH by RIA protocol.

Immunoaffinity on the plate allowed us to observe that the radiolabeled anti-CD20 IgG-SH kept a high affinity for the target antigen (Fig 5). Strong binding (total and specific) was obtained with ^99m^Tc-anti-CD20 IgG-SH, whereas no binding was observed with ^99m^Tc-anti-EGFR IgG-SH (irrelevant antibody). Specific binding of ^99m^Tc-anti-CD20 IgG-SH was confirmed by displacement with an unlabeled antibody. To determine the binding affinity and the maximum number of antibody binding sites/bound per well, a Scatchard analysis was performed. The assays gave an average value of 35 ± 17 nM (n = 3) for the Kd and 11.8 ± 6.5 nM (n = 3) for the binding capacities (B_max_).

**Fig 6:**
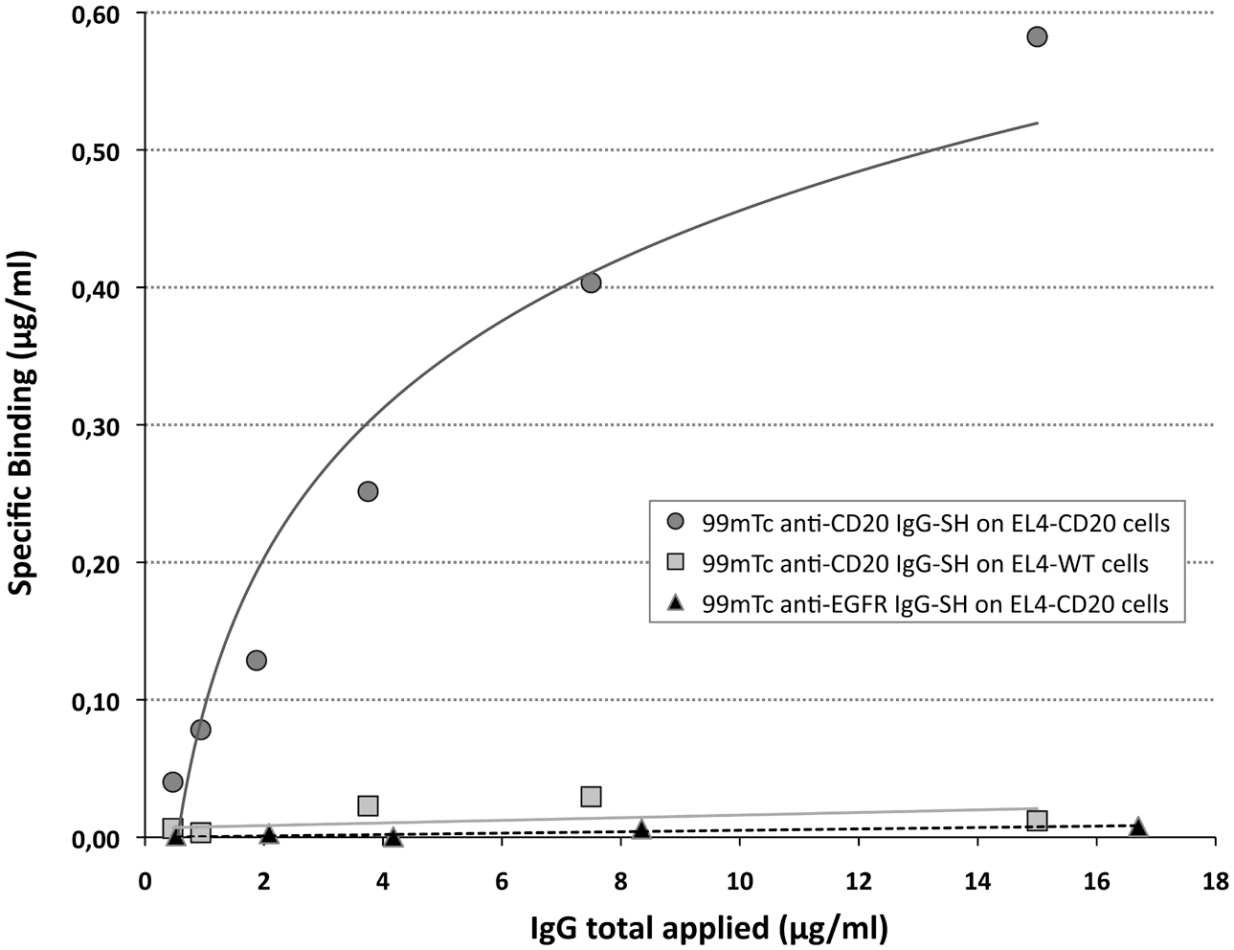
Binding experiments of ^99m^Tc anti-CD20 IgG-SH or ^99m^Tc anti-EGFR IgG-SH on EL4-hCD20 or EL4-WT cells.

Immunoaffinity was also assessed by cell binding on the fully folded antigen expressed on the cell surface. Specifically, radiolabeled anti-CD20 IgG-SH was tested with EL4-hCD20 cells (Fig 6) using a saturation binding assay. The calculated Kd and B_max_ were 40 ± 19 nM (n = 3) and 11 ± 7.2 nM (n = 3), respectively, clearly consistent with the previous value on the plate (Kd = 35 ± 17 nM). Controls included ^99m^Tc-anti-CD20 IgG-SH on EL4-WT cells and with irrelevant IgG, ^99m^Tc-anti-EGFR IgG-SH on EL4-hCD20 cells. As expected, no binding was observed under either condition.

**Fig 7:**
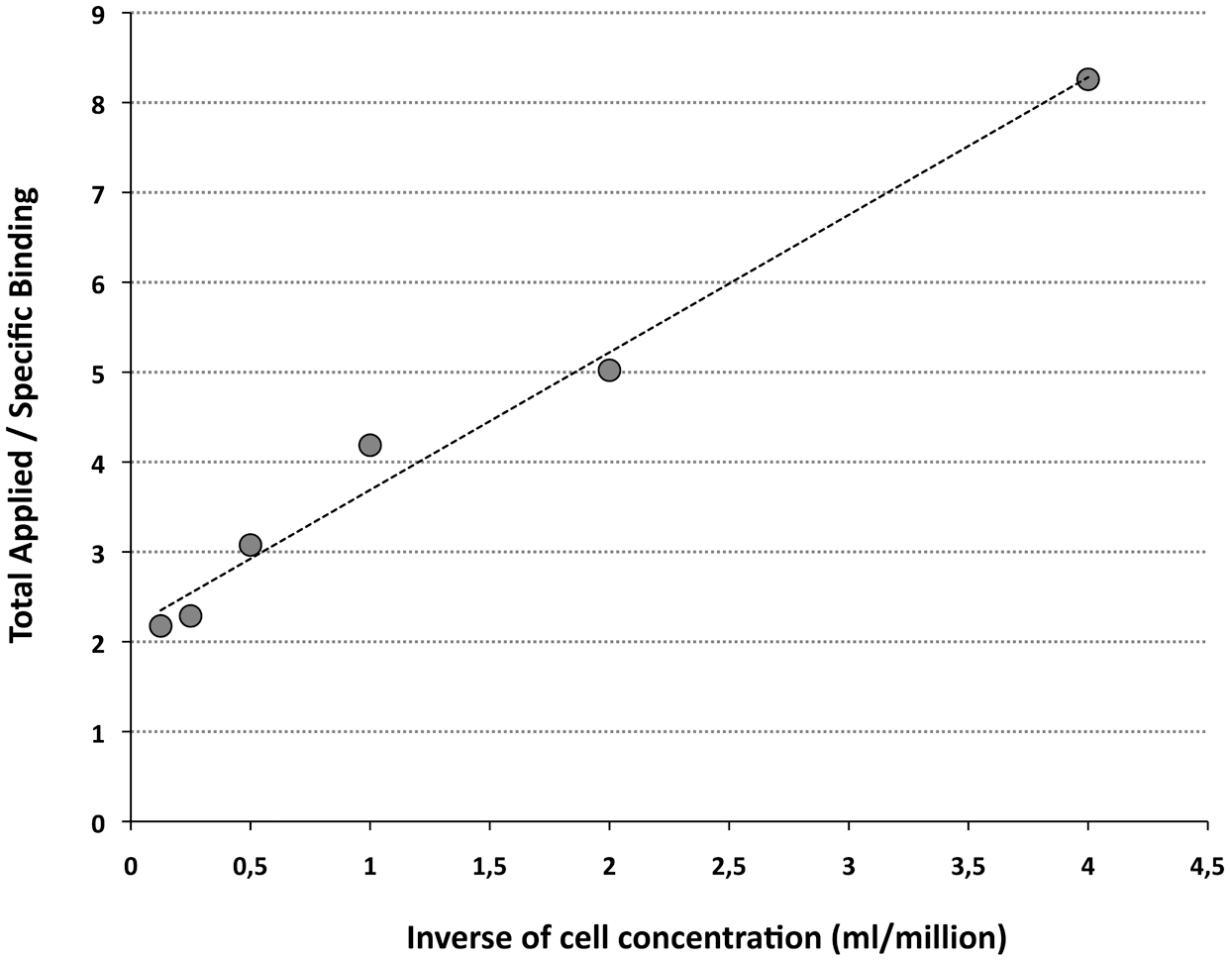
Determination of the immunoreactive fraction (IRF) of ^99m^Tc anti-CD20 IgG-SH. (R^2^ = 0,984).

Finally, to determine the IRF of radiolabeled anti-CD20 IgG-SH, we performed an immunoassay with increasing EL4-hCD20-expressing cell concentrations. Fig 7 shows a plot of total applied radioactivity over specific binding as a function of the inverse of increasing cell concentrations. The IRF can be determined by linear extrapolation. Thus, under our radiolabeling conditions, 46% of the radiolabeled antibodies were able to recognize and to bind specifically with antigens expressed on target cells.

### Biodistribution

The results of the *in vivo* biodistribution of ^99m^Tc-anti-CD20 IgG-SH in healthy BALB/c mice are shown in Table 2. Significant accumulation of radioactivity was observed in four organs as soon as 4h: liver (29.7%ID/g), spleen (27.3%ID/g), lungs (18.0%ID/g) and kidneys (16.7%ID/g). Liver and kidneys radioactivity uptake was related to antibody metabolism and radioactivity elimination. High radioactivity uptake in the spleen and lungs (highly vascularized organs) was correlated with the radioactivity that persisted in plasma at this time (44.0%ID/mL). Different time profiles emerged from these results. A straight decrease profile was observed in plasma (-20% in 14h, and almost -40% in 44h). As expected, spleen and lungs showed the same profile as plasma. An intermediate decrease profile was observed for the liver and kidneys (constant fixation up to 18h). Organs of the digestive tract (stomach, caecum, colon, and small intestine) described a low fixation rate profile (< 5%ID/g) to the five times, indicating that anti-CD20 IgG showed no particular affinity for these tissues which was confirmed by low fixation level in feces (3.6%ID/g at 4h and 1.8%ID/g at 48h). As expected for IgG, the brain showed no significant uptake (%ID/g from 0.6 to 0.2 at 4h and 48h), because IgG does not cross the blood-brain barrier.

**Table 2:** Biodistribution of ^99m^Tc anti-CD20 IgG-SH in healthy BALB/c mice. Values are expressed as the percentage of injected dose per gram (%ID/g) and represent the mean ± S.D. of the %ID/g.

	4h (n = 3)	8h (n = 4)	18h (n = 4)	24h (n = 7)	48h (n = 4)
**Heart**	8,8±2,3	7,7±3,4	7,9±3,2	3,5±1,1	3,7±1,1
**Brain**	0,6±0,2	0,5±0,3	0,5±0,2	0,3±0,1	0,2±0,1
**Lungs**	18,0±1,4	12,0±1,4	8,1±3,2	5,4±1,8	4,8±0,3
**Kidneys**	16,7±3,2	14,8±3,1	16,5±2,9	9,4±2,2	12,3±3,2
**Spleen**	27,3±5,6	17,5±8,6	11,0±3,5	9,7±4,3	9,1±2,5
**Stomach**	3,6±0,7	2,5±0,5	2,7±1,1	0,9±0,4	1,1±0,9
**Small intestine**	3,8±0,8	3,2±1,0	2,9±0,3	1,7±0,4	2,1±0,9
**Cecum**	5,0±1,1	4,2±1,0	3,6±1,7	2,7±1,2	2,1±0,6
**Colon**	3,0±0,5	2,8±0,9	3,6±0,7	1,6±0,4	1,9±0,4
**Liver**	29,7±5,8	22,3±4,3	29,6±11,5	13,6±3,1	15,9±4,3
**Muscle**	1,6±0,5	1,1±0,5	2,9±1,7	1,2±1,0	2,8±1,1
**Blood cells**	4,2±0,5	3,4±0,6	5,2±3,0	1,9±0,8	1,2±0,5
**Plasma**	44,0±7,9	32,4±8,9	28,3±6,9	13,6±3,7	7,6±2,7
**Faeces**	3,6±1,0	3,1±0,9	1,9±1,4	2,2±0,8	1,8±1,8

### Imaging studies

**Fig 8:**
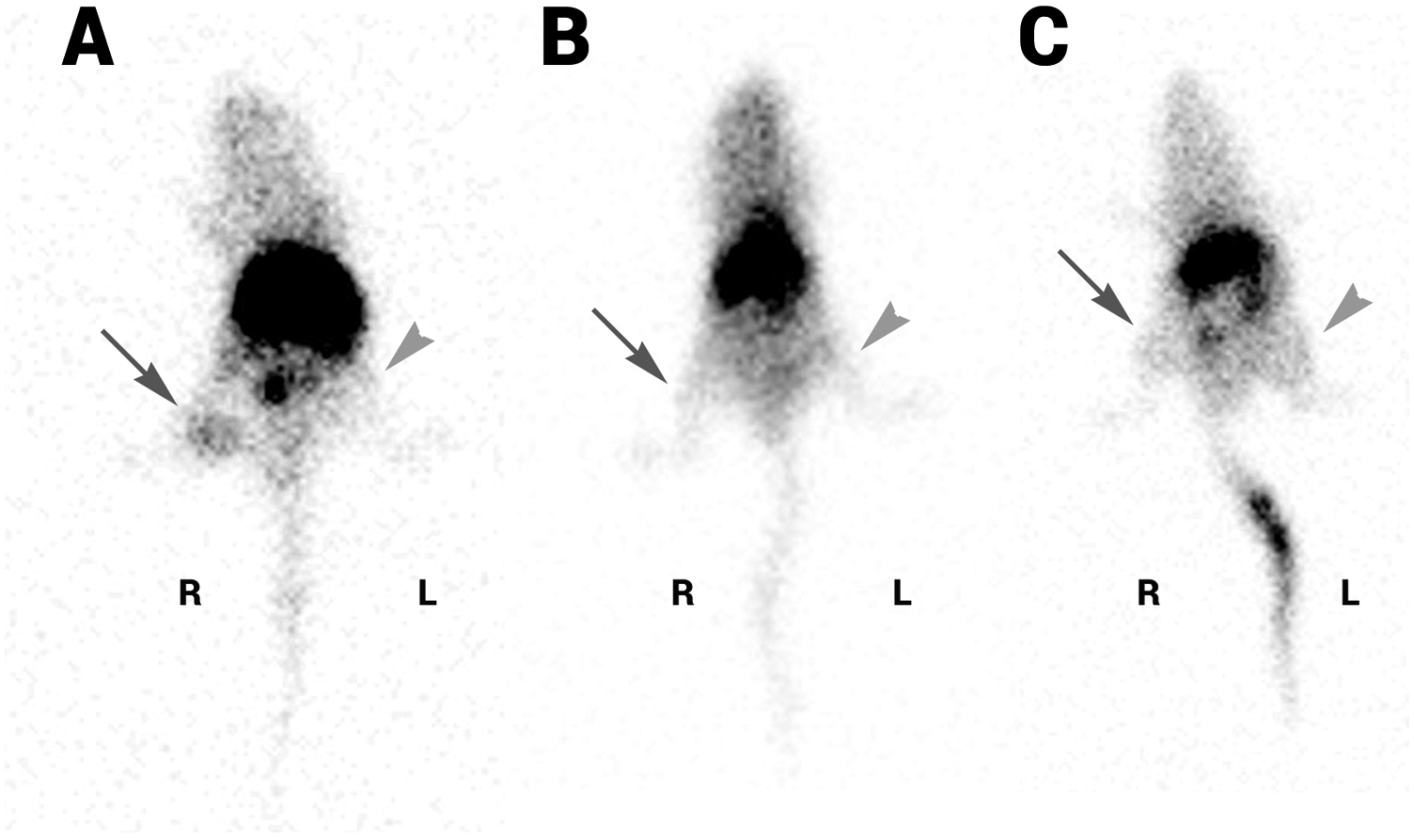
Scintigraphic acquisition of athymic Nude mice bearing CD20+ (R) and CD20- (L) xenografts. Mice were grafted with tumor CD20+ on right flank (R, dark gray arrow) and CD20- on left flank (L, light gray head arrow). Mice were injected intravenously with 35–37 MBq of ^99m^Tc anti-CD20 IgG-SH (A), ^99m^Tc anti-EGFR IgG-SH (B) or ^99m^Tc-anti-IsoLink^®^ (C). Mice were anesthetized with isoflurane (2%, under O_2_ conditions, 1 L/min). Static imaging was acquired, 24h after injection, with a gamma camera (dual-head system fitted with low-energy parallel collimators, 256 × 256 pixels, zoom 2, 10 minutes acquisition).

**Fig 9:**
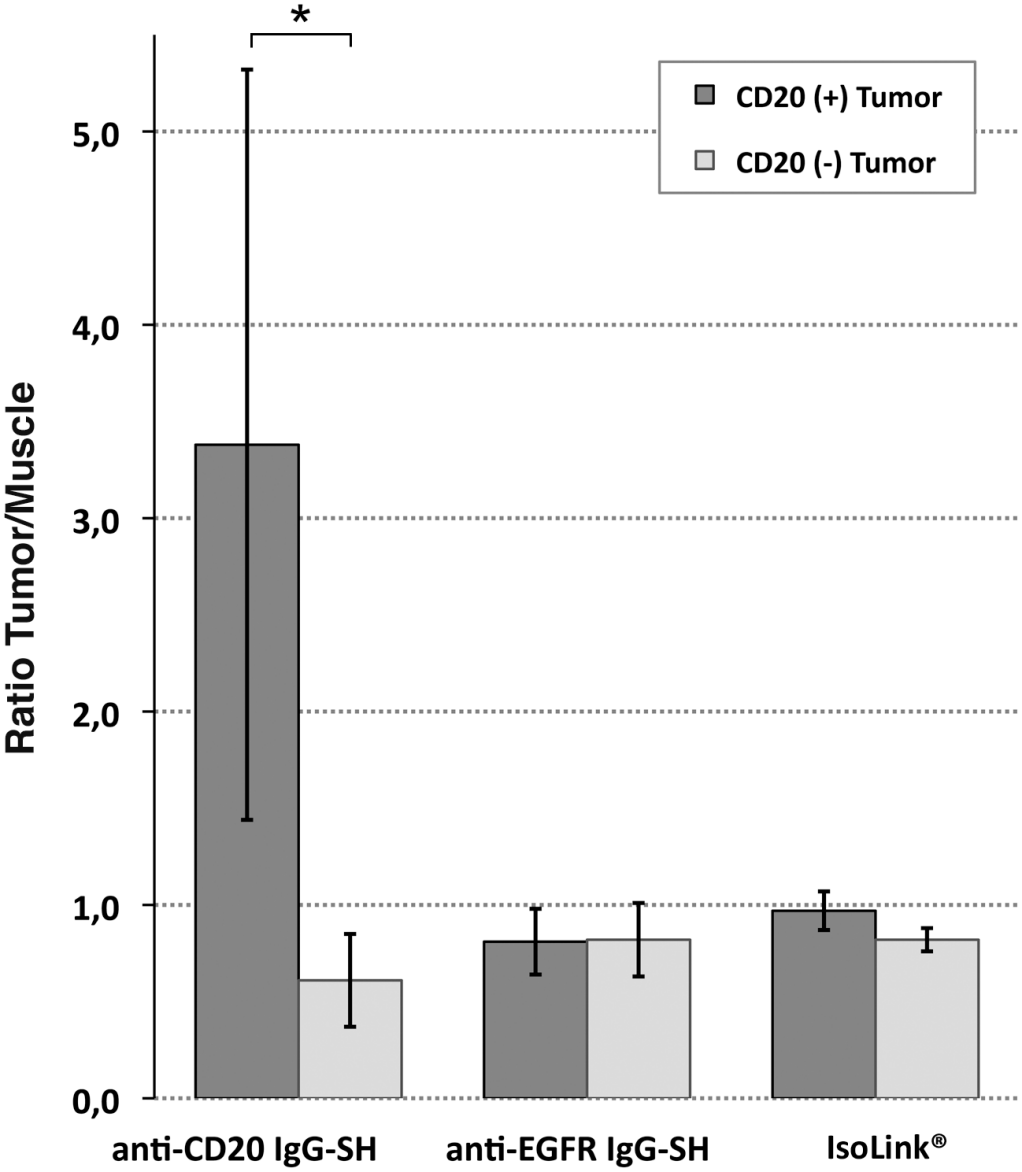
Uptake of ^99m^Tc anti-CD20 IgG-SH, ^99m^Tc anti-EGFR IgG-SH or ^99m^Tc-IsoLink^®^ in CD20+ or CD20- Tumor. Data are expressed as ratio between tumor and normal tissue background (muscle). Significant difference with ^99m^Tc anti-CD20 IgG-SH was observed between CD20+ and CD20- tumor/muscle ratio (*: *p* = 0.018)

To quantify ^99m^Tc-anti-CD20-IgG-SH tumor targeting, nude mice were xenografted with EL4-hCD20 and EL4-WT on the right and left flanks, respectively. ^99m^Tc-anti-CD20 IgG-SH was injected intravenously and planar gamma scintigraphy was performed 2 h 30, 12 h and 24 h after the injection (Table 3, Fig 8A). A fixation was observed in the EL4-hCD20 tumor on the right flank (Fig 8A light gray arrow), whereas no signal was detected in the EL4-WT tumor on the left flank (Fig 8A dark gray arrow). Tumor semiquantitative uptake, normalized by normal tissue background (TBG) uptake in upper limb differed significantly between CD20^+^ tumor and CD20^-^ tumor at each acquisition time. The most striking difference is observed at 24 h (3.38 ± 1.94 versus 0.61 ± 0.24, respectively). High uptake in the liver, spleen and kidneys was also evident, consistent with the biodistribution studies in healthy mice. No fixation was observed in the tumors 24 h after injection of ^99m^Tc-anti-EGFR IgG-SH and ^99m^Tc-IsoLink^®^ (Fig 8B and 8C). For both, “CD20^+^ tumor/TBG” and “CD20^-^ tumor/TBG” ratios between 0.8 and 1 were obtained (Fig 9). No significant difference was observed between CD20+ and CD20- tumors.

**Table 3:** Kinetic uptake of ^99m^Tc anti-CD20 IgG-SH in CD20+ or CD20- Tumor determined by planar scintigraphy. Data are expressed as ratio between tumor and normal tissue background (muscle). Significant differences were observed between CD20+ and CD20- tumor/muscle ratio at 2h30 and 12h (p = 0,03) and at 24 h (p = 0,02).

	2h30	12h	24h
**CD20+ Tumor**	2.58±1.24	3.02±1.22	3.38±1.94
**CD20- Tumor**	0.85±0.62	0.84±0.43	0.61±0.24

## Discussion

This study presents an optimized method to radiolabel anti-CD20 IgG with ^99m^Tc and *in vitro* and *in vivo* evaluations of the radiolabeled IgG. ^99m^Tc is an attractive candidate for radiolabeling due to its specific characteristics: emission, radiation protection, radiochemistry, and ready availability in nuclear medicine department. Due to their high kinetic stability, technetium complexes in a low oxidation state (+I) have recently gained considerable attention for the development of novel site-specific radiopharmaceuticals [15]. The organometallic ^99m^Tc-tricarbonyl core [^99m^Tc(CO)_3_(H_2_O)_3_]^+^ is one of these complexes, characterized by its water- and air-stability. With the IsoLink^®^ kit, this complex can be readily synthesized. This ^99m^Tc-tricarbonyl core is an excellent precursor to radiolabel various biomolecules, especially immunoglobulins. Chen *et al*. [10] radiolabeled native trastuzumab directly with yields of 90%, using commercially available trastuzumab (10 mg). To achieve high synthesis yields with smaller antibody amounts, IgG can be modified before radiolabeling. Dias *et al*. [21] have radiolabeled rituximab with ^99m^Tc-tricarbonyl, after reduction with 2-mercaptoethanol. After disulfide bond reduction, radiolabeling yields reached 98%, but this pretreatment likely induced IgG structural alterations. To functionalize IgG and add SH- groups under milder conditions, Biechlin *et al*. [15] tried an alternative way to derivatize IgG with 2-iminothiolane, and high radiolabeling yields (> 95%) of IgG-SH were obtained with low polyclonal IgG amounts (0.5 mg). To develop a mild radiolabeling method applicable to noncommercial monoclonal IgGs and other isotypes, available only in limited quantities, we selected the method described by Biechlin *et al*. [15]. Our results confirmed the derivatization step’s value: a 40% yield increase was obtained with derivatized IgG versus native IgG (0.0375–0.225mg). A high RCP (> 95%) was achieved with limited IgG amounts (0.150–0.225mg). In comparison, 2 mg of IgG was necessary to reach a RCP of 96% with HYNIC, a standard technology used for protein radiolabeling [22]. As expected, the RCP increased with (the logarithm of) IgG quantity and also with the SH group amount (Fig 3). Under our optimized derivatization conditions, an average of 2.5 moles of sulfhydryl groups was grafted per mole of IgG, compared with 3.3 obtained by Biechlin *et al*. [15] with similar 2-IT concentrations. Moreover, after harsher treatment with 2-mercaptoethanol, Dias *et al*. [21] reached 5 ± 1 SH groups per IgG molecule and obtained RCP > 98%. Radiolabeling is normally qualified by yield, but specific activity is another important parameter. In our experiment, using an average activity of 148–185 MBq, the specific activity of IgG radiolabeled was determinated at 125 MBq/nmol. In comparison, Dias *et al*. [21] reported a lower specific activity of 35 MBq/nmol and Biechlin *et al*. [15] have obtained 80–155 MBq/nmol.

In summary, although we have obtained lower fixation rates of SH groups, radiolabeling yields are comparable to those of other authors, with IgG amount lower and high specific activity. Additionally, antibody immunoaffinity is supposed to be altered less when fewer SH groups are added to binding sites.

^99m^Tc-anti-CD20 IgG-SH radiolabeling stability was checked over 24 h. In PBS, we obtained a decrease of <1% and 3% at 1h and 24h respectively. The comparison of these results with published data using standard method as HYNIC (in saline: <1% and 5% at 1h and 24h respectively) or direct labeling after disulfide bond reduction (decrease of 18% at 22h) showed a better stability of the studied method [22,23]. In plasma, even if our incubation conditions were closer to physiological fluid and harsher than Dias’ or Stopar’s experiments, our stability results were also consistent with published data with tricarbonyl core (loss of 10–15%) [3,21] or direct labeling after disulfide bond reduction [23]. Nevertheless, Singh *et al*. observed with HYNIC a less important release (< 5%) [22]. To challenge tricarbonyl core fixation, displacement experiments with amino acids were conducted and the data obtained with histidine and cysteine were consistent with the results of Dias and Biechlin [21,15]. Cysteine is the most reactive amino acid against the organometallic aquaion [^99m^Tc(CO)_3_(H_2_O)_3_]^+^. As described by Dias [21], we observed that a 1500-fold excess was necessary to decrease the RCP to 9%. With HYNIC method, the reduction of the labeled IgG was quite similar to our study: at 1h, even at a molar ratio of 25 times, the decrease was about 5.8%, which increased to 23.7% at 100 times cysteine concentration [22]. Study with MAG3 method gave same result (dissociation of ^99m^Tc of 18% for an excess of 50) [24].

Furthermore, our optimized radiolabeling process, as observed by Western blot, did not impair the native IgG structure. Few integrity studies have been reported, although protein degradation was described for polyclonal IgG radiolabeled after stannous or 2-mercaptoethanol reduction [24]. This result was consistent with the theoretical advantage of the BFCA method, which should be milder than reduction.

The last step to investigate radiolabeled antibody functionality was to perform binding studies. Two parameters were determined: the dissociation constant and the immunoreactive fraction. Results obtained for Kd with the two methods, on a plate and on cells, were consistent and complementary. The technique on the plate is easy to conduct and provides an approximation of affinity, but variation was observed probably due to inconsistent antigen adsorption on the support. Experiments with cells, are closer to *in vivo* conditions in terms of antigen presentation. Moreover, the same cell lines can then be grafted to induce experimental tumors. However, for a selected cell line, the number of binding sites depends on culture conditions (e.g., the number of cells transplanted, cells confluency states, cell cycle phase). In both experiments, we determined a Kd of ~40 nM, which represents a small loss of affinity compared with native anti-CD20 IgG (Kd = 5.2–11.0 nM) [16]. This slight affinity loss can be explained both by the derivatization with 2-IT and by radiolabeling with the tricarbonyl core. The IRF obtained (46%) indicates that only a portion of the radiolabeled antibodies can bind to antigen, which likely causes background noise during image acquisition. However, the IRF value is close to those obtained in other studies on rituximab radiolabeled with the tricarbonyl core. Surprisingly, despite a good Kd, Dias *et al*. [21] determined a similar IRF of 50%.

The results of the biodistribution studies carried out in normal BALB/c mice with ^99m^Tc-anti-CD20 IgG-SH were consistent with reported values [11,21]. Thus, high uptake of radioactivity into the lungs and the spleen was observed with IgG and can be explained by the high plasma radioactivity and by the general characteristics of radiolabeled IgG: high initial activity levels in blood and blood-rich tissues [25]. High activity retention in the liver and in kidney can then be attributed to hepatobiliary metabolism of the radiolabeled antibody and renal clearance of the degraded form.

Rituximab is characterized by a half-life of ~3 weeks (17–21 days). Activity in plasma, which remained high, is correlated with this relatively long biological IgG half-life in the bloodstream, which could explain why radioactivity is still present in plasma after 48 h post-injection (10%), and suggests the absence of rapid degradation.

Preliminary imaging studies performed in animals confirmed the *in vitro* affinity results. Mice bearing subcutaneous lymphoma expressing CD20 on the right side and wild type on the left side showed a significant uptake in CD20^+^ tumor versus CD20^-^ tumor This favorable uptake of rituximab in the CD20^+^ tumor is comparable with the uptake obtained by Dias *et al*. [21] with a tumor model of a Ramos lymphoma xenograft (CD20^+^ tumor/muscle = 4.0).

## Conclusions

The association of a bifunctional synthon (2-iminothiolane) and a preformed technetium complex (^99m^Tc-tricarbonyl) made it possible to achieve high radiolabeling yields, with high specific activity. Additionally, the radiolabeled IgGs were stable in PBS and in plasma. Their structure and immunoaffinity were not impaired by this method. Specific anti-CD20 binding was conserved both *in vitro* and *in vivo*. Small antibody amounts can be used with our optimized radiolabeling method. This major advantage could be exploited to radiolabel antibodies expensive or available in very limited quantities, such as different Ig isotypes or antibody fragments.
